# An individual’s trust in government is a major determinant in the decision to voluntarily join a public health initiative

**DOI:** 10.1186/s13584-025-00671-x

**Published:** 2025-02-14

**Authors:** Orit Golan, Carmit Satran, Shiran Bord

**Affiliations:** 1 Department of Health Systems Management, The Max Stern Yezreel Valley College, Yezreel Valley, 1930600 Israel; 2 Department of Nursing, The Max Stern Yezreel Valley College, Yezreel Valley, 1930600 Israel

**Keywords:** Public health, Trust, Perceived threat, Digital surveillance technologies, Policy, Covid-19

## Abstract

**Background:**

Recently, there has been a growing trend of incorporating technology using health applications by official organizations such as health organizations and governmental bodies. In response to the COVID-19 pandemic, Israel implemented a health application to be voluntarily downloaded by citizens (VA). Additionally, the Israeli authorities used mandatory mobile tracking to monitor citizens’ movements (GT). The current study aims to identify the factors associated with individuals’ decisions to download the voluntary contact-tracing app. We hypothesized that (a) attitudes toward GT will mediate the relations between trust in the healthcare system and downloading VA, and (b) attitudes toward GT will mediate the relations between the perceived COVID-19 threat and downloading VA.

**Methods:**

Data were collected among 741 respondents who completed an online survey on July 19–21, 2020. The survey was designed to represent the diversity of the Israeli population. A logistic regression was calculated with downloading VA as the dependent variable and trust in the healthcare system, perceived threat and attitudes toward GT as independent variables. Then, the extent to which attitudes toward GT mediated the associations between trust in the healthcare system and downloading VA and between the perceived threat and downloading the voluntary app was assessed using the Process procedure.

**Results:**

The findings reveal that 47.1% of respondents perceive governmental tracing as an invasion of privacy, while 24.4% report that it increased their sense of security. About a third report having downloaded the voluntary app. Both research hypotheses were supported; attitudes played a mediating role in the relationship between an individual’s level of trust and an individual’s level of perceived threat and behavior, i.e., higher trust and higher perceived COVID-19 threat were associated with more favorable attitudes towards GT, which was associated with more substantial odds of downloading VA.

**Conclusions:**

The results emphasize the crucial importance of public trust. Building trust with the public is essential to encourage voluntary actions that ultimately benefit public health. Achieving and maintaining the public’s trust requires addressing concerns about the potential misuse of government power and fostering an environment that promotes voluntary participation and engagement.

**Supplementary Information:**

The online version contains supplementary material available at 10.1186/s13584-025-00671-x.

## Introduction

The COVID-19 virus surprised the entire world with its intensity and dissemination speed. Each country adopted a different strategy to fight the virus and slow the rate of infection and mortality (“flatten the curve”). Thus, initially, countries had to formulate an immediate comprehensive action plan that simultaneously included treating illness and preventing infections and the spread of the disease. When formulating the plan, each country had to consider the need to protect public health and individual human rights and strike a balance between the two.

To break the chains of infection and mitigate contagion, many governments developed digital contact-tracing applications for epidemiological investigation purposes. In most countries, these tools were invoked by encouraging people to use voluntary applications (VA) developed during the crisis to help detect contacts and weaken chains of infection [1]. While digital contact tracing offered promising tools for controlling the virus spread, it also raised fundamental questions regarding the balance between public health needs and civil liberties. Countries faced complex decisions about whether to rely on voluntary cooperation through downloadable apps or implement mandatory tracking measures. These choices reflected not only technological capabilities but also deeper societal values regarding privacy, individual rights, and the government’s role in public health emergencies.

In Germany, the federal government and the Centers for Disease Control and Prevention (CDC) launched their official COVID-19 contact tracing VA, Corona-Warn-App, on June 16, 2020 [2]. This VA was an open-source Bluetooth-based decentralized smartphone app employing a privacy-preserving model, collecting anonymized contact data stored locally on the user’s smartphone [3].

In March 2020, the government in Singapore asked citizens to install a government-developed smartphone VA called Trace Together. This app uses Bluetooth technology to exchange identifier numbers with the phones of other Trace Together users within 6 feet of the user, sharing data with the government only if the user becomes subject to contact tracing due to a COVID-19 diagnosis [1, 4].

The VA in Belgium was developed to provide cellular communication without revealing personal information or locations. This app traces contact with COVID–19–diagnosed individuals, notifying app users of this contact without revealing the identity of the diagnosed app user(s) and where this contact occurred [5].

In the United Kingdom, the National Health Service (NHS) launched a VA in September 2020 [1]. The technology for this app is based on the Google Apple Exposure Notification (GAEN) system. The data are stored in the app for 14 days only. When a user tests positive for COVID-19, the app requests consent to have the information sent anonymously to a central server.

In Israel, on March 16, 2020, the government approved emergency regulations allowing mass location tracking of citizens as part of the national effort to slow the spread of the pandemic [6]. These regulations served two purposes: enforcing quarantine rules (with violation of quarantine possibly resulting in the issuance of a fine) and tracking the locations of those infected with the virus. In fact, Israel used two digital contact-tracing methods simultaneously: governmental tracing (GT), operated by the Israel Security Agency (ISA), and a GPS-based VA (the Protector app), which traces contacts by using GPS and is subject to individuals’ choice. Hence, Israel was one of the few democracies that used GT to track potential contacts with infected individuals. Notably, the ISA’s primary mission lies elsewhere: to thwart terrorism and espionage.

In addition to Israel, two examples of countries that opted for mandatory GT supervision of citizens are South Korea and China. In South Korea, geolocation data were used without seeking consent. The government publicly posted information regarding infected persons’ whereabouts in the days before their diagnosis, basing its reportage on cellphone location data, credit card records, and surveillance videos. No names were included, but individuals’ age, nationality, and gender were disclosed [1, 4].

In China, the government required citizens in more than 200 cities to install an Alipay app on their smartphones, assigning each person a risk code indicating how and where they are permitted to move around the community. The coding algorithm reportedly includes information on time spent at risky locations and frequency of contact with other people [1].

A significant concern is that addressing a global health crisis may amplify governments’ ability to make decisions that could ultimately harm citizens’ rights, allowing them to accumulate power and use or abuse it without restraint or oversight [7]. The World Health Organization [8] appears to have identified this challenge in the first stages of the pandemic. Therefore, its Director General, in his opening remarks at a mission briefing on COVID-19 on March 12, 2020, declared: “All countries must strike a fine balance between protecting health, preventing economic and social disruption, and respecting human rights.”

To attain this balance, the Supreme Court of the United States holds that a judicial warrant must be obtained to search private cellphone records for law-enforcement purposes after probable cause to believe a person violated the law is shown [1]. This limits the government’s ability to use GT applications.

The CDC launched a decade-long public deliberation regarding the ethical use of public health data collection, including surveillance [9]. Since then, peer-reviewed literature and guidance documents have outlined the ethical foundations of public health surveillance [10, 11]. These documents emphasize the importance of using public health surveillance data for public health purposes only, assuring the privacy and confidentiality of surveillance data, collecting the least data necessary to achieve the public health objective, building trust and engaging affected communities [12].

As the scientific literature indicates, most Western countries used VA rather than mandatory GT. The success of VA, however, critically depends on people’s willingness to use these apps. Hinch and his colleagues showed that using tracing apps in the UK mitigates infections at all levels but suffices in stopping the epidemic only if used by approximately 60% of the population [13]. Therefore, it is essential to gauge the strength of public support for this approach and understand the factors that may hinder or facilitate uptake.

Public attitudes towards COVID-19 digital contact-tracing applications play a pivotal role in their adoption and effectiveness. Studies highlight that trust in institutions, privacy concerns and social responsibility are key determinants shaping public perceptions of these technologies. Altruistic motivations, such as protecting others and mitigating the virus spread, also encouraged uptake despite reservations [14, 15]. Praveen and Ittamalla found that 49% of the general public’s sentiments regarding governments using digital contact tracing to manage COVID crises were neutral. In comparison, 29.9% (*n* = 1504) expressed positive sentiments and 20.8% (*n* = 1050) expressed negative sentiments [16]. The importance of an individual’s attitude is well described by Ajzen’s and Fishbein’s Theory of Reasoned Action (TRA) [17, 18]. As far as we know, TRA components have not been studied directly in relation to the use of digital tracing during the COVID-19 pandemic. They have, however, been found relevant when used to analyze voluntary self-isolation behavior during COVID-19 in Spain and Colombia [19]. An Israeli study [20] analyzed public discourse, as reflected in media outlets, regarding the use of digital surveillance technologies for monitoring population behavior during the COVID-19 pandemic. The study revealed that there was significant opposition to the deployment of these digital surveillance methods. People were concerned about the potential for these technologies to lead to authoritarianism and infringement of fundamental rights. Many posts and comments showed that people were worried about the dangers associated with these technologies.

Cultural and sociodemographic factors, such as collectivism, education, and age, influenced attitudes, with public acceptance varying significantly across contexts [21]. Although attitudes are not always explicitly analyzed as mediating variables, they are evident intermediaries linking external factors, such as trust and privacy concerns, to decision adoption [9, 22].

Multinational anonymous online surveys conducted by Altmann and his colleagues in France, Germany, Italy, the UK and the US demonstrate an association between citizens’ willingness to voluntarily download digital tracing apps and share their data with the government, and their level of trust in the government [4]. According to this study, while the willingness to install these apps is high and the available evidence suggests that app-based contact tracing could be an effective method for controlling the spread of COVID-19, concerns regarding privacy and cybersecurity, together with a lack of trust, continue to be significant barriers to downloading a VA. For instance, trust in healthcare institutions positively influenced app adoption, while distrust in governments and private companies, coupled with privacy concerns, posed significant barriers [23, 24]. Misconceptions regarding app functionalities, including fears of surveillance or data misuse, were prevalent and often exacerbated skepticism [21,22).

Larson and her colleagues define trust as “a relationship between individuals, as well as between individuals and a system, in which one party accepts a vulnerable position, assuming the best interests and competence of the other, in exchange for a reduction in decision complexity” (p.1599) [25]. Trust appears to be a critical component of public response to emergencies [26, 27]. A low level of trust in the healthcare system is associated with poor compliance with healthcare guidelines [27–29].

A recent study [3] found that trust in the app’s perceived security and belief in its effectiveness are key psychological factors influencing its adoption. Trust significantly impacted past decisions to download the app, while belief in its effectiveness was crucial for future download intentions. This may also relate to the perceived threat of the disease, as the decision might reflect a balance between trust levels and the intensity of the perceived threat.

While trust in healthcare systems and the perceived threat can independently influence behavior, their interaction, particularly regarding attitudes toward surveillance technologies, plays a vital role in voluntary app adoption.

According to the Health Belief Model (HBM) [30], the term “perceived threat” combines perceived vulnerability to a given disease and the perceived severity and/or consequences of this disease. According to a study which explored the perceived threat level of COVID-19 in 10 countries in Europe, Asia and the Americas [31], the public’s perceived threat was generally high, but lower when the participants had higher levels of trust in the government. Several Israeli studies which assessed the perceived threat of COVID-19 among various population groups revealed an overall high level of perceived threat [29, 32, 33].

The theoretical framework integrating trust, perceived threat and attitudes toward surveillance technologies suggests a complex pathway to voluntary app adoption. According to the Theory of Reasoned Action (TRA), attitudes serve as crucial mediators between individuals’ beliefs and their behavioral intentions. In the context of digital contact tracing, both trust in the healthcare system and the perceived COVID-19 threat may shape individuals’ attitudes toward governmental tracking, which in turn could influence their decision to download voluntary apps. Trust may foster more accepting attitudes toward governmental surveillance by increasing confidence in the authorities’ proper use of tracking data. Similarly, higher perceived threat may lead to more favorable views of tracking measures as necessary safety precautions. Rather than trust or threat perceptions alone, these attitudes may be the proximal determinants of individuals’ willingness to download contact tracing apps. This mediation framework is particularly relevant in the Israeli context, where mandatory governmental tracking coexisted with voluntary applications, providing a unique setting for examining how attitudes toward imposed surveillance measures might influence voluntary technological adoption for public health purposes.

In light of the above, the current study aimed to identify the factors associated with individuals’ decision to download a voluntary COVID-19 tracing app. More specifically, it was designed to examine the mediating role of the public’s attitudes towards GT in the association between public trust in the healthcare system and VA downloading, as well as in the association between the perceived COVID-19 threat and VA downloading. We hypothesized that (a) attitudes toward GT will mediate the relations between trust in the healthcare system and downloading VA, and (b) attitudes toward GT will mediate the relations between the perceived COVID-19 threat and downloading VA.

The literature review suggests that these mediation models have not previously been tested. Additionally, as GT has not been extensively utilized, the current study offers a distinctive context for examining these hypotheses.

## Materials and methods

We analyzed data collected among 741 respondents who completed an online anonymous survey on July 19–21, 2020. The survey, managed by a well-known large online survey company (iPanel), was designed to represent the diversity of the Israeli population. Prior to filling out the survey questionnaire, participants were required to give their consent by answering a yes or no question.

### Participants

The participants were 741 Israeli adults aged 18–72. Their average age was 40 (Table 1). Approximately half were females, 74.8% were Jewish (mostly Israel-born), and 46.7% were secular. Approximately 60% were married or in an intimate relationship; most of the others were single. Approximately 61% had children, and around 45% had minor children. Approximately 42% had an academic education, and most of the others had a high-school education (26.8%) or higher (21.4%). Most of the respondents (73%) were employed at the time of the survey, and 55.2% reported below-average income.

**Table 1:** Participants’ socio-demographic characteristics (*N* = 741)

Age (years) M (SD)	18–72	38.10 (13.77)
Gender n(%)	Female	381 (51.4)
Religion n (%)	Jewish	554 (74.8)
	Muslim	124 (16.7)
	Christian	39 (5.2)
	other	24 (3.3)
Country of birth n (%)	Israel	661 (89.2)
Marital status n (%)	Married, in an intimate relationship	452 (61.0)
	Single	223 (30.1)
	Divorced, separated, widowed	66 (8.9)
Having children n (%)	Yes	451 (60.9)
Having minor children n (%)	Yes	331 (44.7)
Number of children *M* (*SD*)	1–13	2.86 (1.67)
Education level *N* (%)	High school	198 (26.8)
	Higher education	158 (21.4)
	Student	69 (9.3)
	Academic	314 (42.5)
Current employment status *N* (%)	Employed	536 (73.0)
	Unemployed due to Covid-19	64 (8.7)
	Unemployed pre Covid-19	45 (6.1)
	Unemployed - other (student, retired)	90 (12.2)
Income level *N* (%)	Below average	406 (55.2)
	Average	211 (28.7)
	Above average	118 (16.1)
Religiosity *N* (%)	Secular	346 (46.7)
	Partly religious	213 (28.7)
	Religious	182 (24.6)

Percentages were calculated excluding missing data

### Tools and measures

The study was designed to examine the associations between VA downloading and perceived COVID-19 threat, trust in the healthcare system and attitudes toward the use of GT.

The questionnaire was constructed on the basis of several valid questionnaires in Hebrew [29, 32] and English [18, 30]. The items on the English-language questionnaires were translated into Hebrew and then back-translated into English to ensure the reliability of the Hebrew questionnaire. The research protocol was approved by the ethics committee of the investigators’ academic institution (Ethics Number Emek YVC 2020-94).

#### Dependent variable: voluntary downloading of the protector app—VA

VA was measured using the question: “Have you downloaded the Protector app to your cell phone?” (yes/no).

#### Perceived COVID-19 threat

To measure the perceived COVID-19 threat, we used the HBM scale of perceived threat [30], which combines perceived vulnerability and severity. Nine items were adapted to match the perceived risk of COVID-19 to the respondents and their significant others, the perceived severity of COVID-19, and the perceived risk to the individual’s financial well-being (Cronbach’s α 0.76). For example: “The thought that I will get sick with COVID-19 scares me”; “If I get sick with COVID-19, it will be difficult for me to keep functioning”; “If I get sick with COVID-19, it will be a more severe disease than other diseases.” Respondents were asked to rate each item on a 5-point Likert scale, from 1 (strongly disagree) to 5 (strongly agree). Mean scores were calculated for each respondent, with higher total scores representing higher perceived COVID-19 threat.

#### Trust in the healthcare system

Trust in the healthcare system was measured using a 5-item subscale of the Multidimensional Trust in Healthcare Systems Scale (MTHCSS), developed by Egede and Ellis [34]. Examples of items are: “The Israeli healthcare system is doing its job well”, and “I trust the medical information published by the Israeli healthcare system.” Respondents were asked to rate each item on a 7-point Likert scale from 1 (strongly disagree) to 7 (strongly agree). Cronbach’s α was 0.77 for the five items of the trust scale. Mean scores were calculated for each respondent, with higher total scores representing greater trust in the healthcare system.

#### Attitudes toward GT

Attitudes toward GT were measured using four items based on the attitude subscale developed by Ajzen and Madden [18] and adjusted for the current study. Examples of items are: “The ISA’s cellular tracing is justified in light of the COVID-19 pandemic” and “I believe that during the COVID-19 pandemic, it is necessary to follow people to prevent contagion.” Respondents were asked to rate each item on a 7-point Likert scale, from 1 (strongly disagree) to 7 (strongly agree). Cronbach’s α was α = 0.82 for the four items, with a higher score reflecting more positive attitudes toward GT.

#### Covariates

Respondents were asked to provide the following details about themselves: age, gender, ethnicity (Jewish, Arab), religiosity (secular or partly religious vs. ultra-religious), marital status (married or in an intimate relationship vs. unmarried), having children (yes/no), education level (non-academic, academic), employment (yes/no), and income level (below average, average or above average).

### Statistical analysis

The data were analyzed using SPSS ver. 27. There were no missing data for the research variables and major demographic variables. Internal consistencies were examined, and variables were composed based on item means. Demographic differences between respondents who downloaded VA and those who did not were calculated using Z tests to determine the significance of the difference between two independent proportions. The research variables were described in terms of means, standard deviations and Pearson correlations. A multiple hierarchical regression was calculated for attitudes toward GT as the dependent variable, and trust in the healthcare system and perceived threat as independent variables. Next, logistic regression was calculated with downloading VA as the dependent variable and trust in the healthcare system, perceived threat and attitudes toward GT as independent variables. Finally, the extent to which attitudes toward GT mediated between (a) trust in the healthcare system and downloading VA, and (b) perceived threat and downloading VA, was assessed using the Process procedure [35], Model 4, twice, for the binary dependent variable, using 5,000 bootstrap samples and a 95% confidence interval. As there were two hypotheses with two independent variables, two Process models were calculated. Covariates were the same as in the logistic regression model: ethnicity, religiosity and family status. P value was set at 0.05.

## Results

### Descriptive results

About one-third of the respondents (*N* = 270, 36.4%) downloaded the VA. Table 2 compares their background characteristics with those of respondents who had not downloaded the app. The table reveals several differences, the major of which concerns religiosity: the rate of VA download was significantly higher among secular and partly religious respondents than among religious respondents. Other differences were smaller yet still significant. Arab respondents downloaded VA more than Jewish ones, non-married respondents more than married ones, and respondents without children more than those with children. In addition, unemployed respondents downloaded VA more than employed ones.

**Table 2:** Downloading the Voluntary tracing app by participants’ demographic characteristics (*N* = 741)

		Yes (*n* = 270) *n* (%)	No (*n* = 471) *n* (%)	*P*
Gender	Male	127 (35.3)	233 (64.7)	0.524
	Female	143 (37.5)	238 (62.5)	
Ethnicity	Jewish	190 (34.3)	364 (65.7)	0.037
	Arab	80 (42.8)	107 (57.2)	
Religiosity	Secular, partly religious	224 (40.1)	335 (59.9)	< 0.001
	Religious	46 (25.3)	136 (74.7)	
Marital status	Married, in an intimate relationship	151 (33.4)	301 (66.6)	0.032
	Not married	119 (41.2)	170 (58.8)	
Having children	Yes	151 (33.5)	300 (66.5)	0.037
	No	119 (41.0)	171 (59.0)	
Education level	Not academic	121 (34.0)	235 (66.0)	0.166
	Academic	149 (38.9)	234 (61.1)	
Employment	Yes	181 (33.8)	355 (66.2)	0.013
	No	87 (43.7)	112 (56.3)	
Income level	Below average	153 (37.7)	253 (62.3)	0.444
	Average and above	115 (35.0)	214 (65.0)	
Age	18–72	M = 37.64 (SD = 14.13)	M = 38.37 (SD = 13.56)	0.486

Table 3 presents the distribution and inter-correlations of the study variables. Means for trust, perceived threat, and attitudes toward GT were moderate. Few outliers were noted in these variables (in up to 1% of the cases, different cases in two of the independent variables), and no deviations from a normal distribution were noted (skewness = -0.20 to -0.55, SE = 0.09). Positive low to moderate correlations were found among trust, perceived threat and attitudes toward GT. The last was positively associated with downloading the VA.

**Table 3:** Means, standard deviations and inter-correlations of study variables (*N* = 741)

	M (SD)	Trust in the healthcare system	Perceived threat	Attitudes toward GT	Downloading VA
Trust in the healthcare system (1–7)^a^	4.18 (1.09)	1			
Perceived threat (1–5)^b^	3.51 (0.58)	0.15***	1		
Attitudes toward GT (1–5)^b^	3.06 (0.90)	0.35***	0.26***	1	
Downloading VA (0–1)^c^	0.36 (0.48)	0.01	0.09*	0.29***	1

*P <.05, **P <.01, ***P <.001

^a^scale ranges from 1 = strongly disagree to 7 = strongly agree

^b^scale ranges from 1 = strongly disagree to 5 = strongly agree

^C^0 = no; 1 = yes

### Attitudes toward GT

Almost half of the respondents (47.1%) perceived GT as an invasion of their privacy (M = 5.06, range 1–7), but 24.4% reported that this governmental measure increased their sense of security (M = 2.70, range 1–5). Furthermore, 48.4% agreed or strongly agreed with the statement that the government uses the data gathered for other purposes (not only for fighting the pandemic—M = 3.36, range 1–5).

Attitudes toward GT were generally not associated with respondents’ demographic characteristics. The sole exception was ethnicity: Jewish participants reported more positive attitudes toward GT (M = 3.12, SD = 0.87) than did Arab participants (M = 2.88, SD = 0.95) (t (739) = 3.28, *P* =.001).

### Downloading VA

Downloading VA is found to be associated with ethnicity, religiosity, marital status, having children and employment status (Table 2). A logistic regression was performed to assess how trust in the healthcare system, perceived threat and attitudes toward GT were associated with downloading the app. The control variables were ethnicity (1-Jewish, 0-Arab), religiosity (1-secular or partly religious, 0-religious), and family status (1-married or in an intimate relationship, 0-unmarried). Having children was not used due to its strong association with family status (phi = 0.63, *P* <.001), and employment status was not used due to its low variance (73% employed). Table 4 presents the logistic regression. No collinearity was noted (highest VIF value = 1.21, tolerance = 0.82), and the Box-Tidwell test supported the linearity of the independent variables and the log odds. The results show that beyond socio-demographic variables, perceived threat and positive attitudes toward GT were associated with higher odds of downloading VA.

**Table 4:** Logistic regression for downloading of Voluntary tracing app (*N* = 741)

	*B*	*SE*	*OR* (95%CI)	Nagelkerke *R*^2^
Step 1				0.03***
Ethnicity	-0.28	0.18	0.76 (0.54, 1.07)	
Religiosity	0.63	0.19	1.88 (1.29, 2.75)***	
Family status	-0.24	0.16	0.78 (0.57, 1.07)	
Step2				0.17***
Ethnicity	-0.50	0.19	0.61 (0.42, 0.88)**	
Religiosity	0.73	0.20	2.08 (1.39, 3.10)***	
Family status	-0.22	0.17	0.80 (0.57, 1.12)	
Trust in the healthcare system	0.01	0.07	1.02 (0.88, 1.17)	
Perception of threat	0.34	0.14	1.41 (1.07, 1.85)*	
Attitudes toward use of governmental tracing	0.82	0.10	2.27 (1.85, 2.79)***	

*P <.05, **P <.01, ***P <.001; χ^2^(6) = 99.44, *P* <.001

**Fig. 1:**
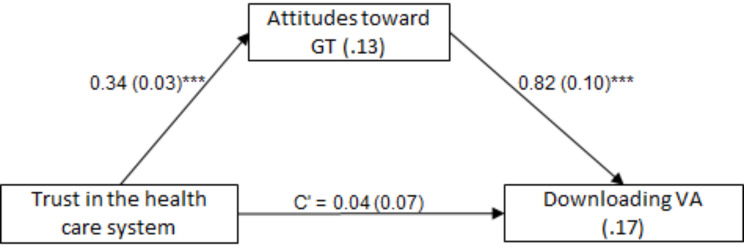
The mediating role of attitudes toward GT in the association between trust and downloading VA. *Note* Values on arrows: B(SE), values within rectangles: R^2^ and Nagelkerke’s R^2^, C’ = direct effect. *p <.05, **p <.01, ***p <.001

**Fig. 2:**
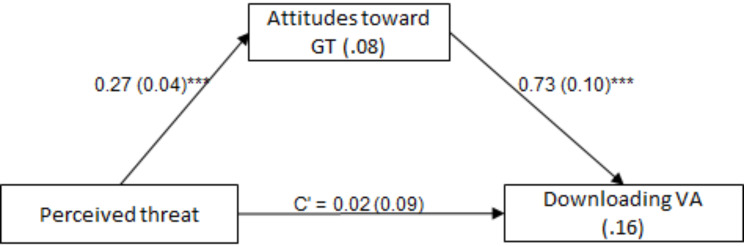
The mediating role of attitudes toward GT in the association between perceived threat and downloading VA. *Note* Values on arrows: B(SE), values within rectangles: R^2^ and Nagelkerke’s R^2^, C’ = direct effect. *p <.05, **p <.01, ***p <.001

To determine whether attitudes toward GT mediate relations between (a) trust in the healthcare system and downloading VA, and (b) perceived threat and downloading VA, the Process procedure [35], Model 4, was used twice, with 5,000 bootstrap samples and a 95% confidence interval. The indirect effects in both models were significant: for trust in the healthcare system - effect = 0.28, SE = 0.05, Z = 5.82, *P* <.001, 95%CI = 0.20, 0.38; and for perceived threat - effect = 0.20, SE = 0.04, Z = 4.86, *P* <.001, 95%CI = 0.13, 0.28. That is, higher trust in the healthcare system was associated with more favorable attitudes toward GT, which was associated with stronger odds of downloading VA. Further, higher perceived threat was associated with more favorable attitudes toward GT, which was associated with stronger odds of downloading VA (Figs. 1 and 2, Supplementary Table 1). Both research hypotheses were supported.

## Discussion

This study examined the mediating role of attitudes toward governmental tracking (GT) in the association between trust in the healthcare system, perceived COVID-19 threat, and voluntary app (VA) downloading. The findings support our theoretical model, demonstrating that attitudes toward GT are a critical mediating mechanism. This mediation effect aligns with the Theory of Reasoned Action’s emphasis on attitudes as key determinants of behavioral intentions, particularly in the unique Israeli context where mandatory and voluntary tracking measures coexisted. This context provides unique insights into how attitudes towards surveillance technologies shape public health behaviors. The lower adoption rate of voluntary apps in Israel, as compared to other countries, may reflect the complex interplay between mandatory and voluntary surveillance measures.

While using digital epidemiology tools has the potential to mitigate infections [5], it may also raise ethical questions and ignite debates [1]. The Israeli Democracy Institute underscores the critical role of digital tracing in mitigating infections but also emphasizes the essential need to consider the cost to the public’s privacy, even for the sake of protecting public health [36].

In a democracy, voluntary cooperation and compliance with mandatory regulations depend mainly on public trust in policymakers and the healthcare system [37, 38]. Moreover, in times of severe threat, people may be willing to surrender some of their privacy and freedom to maintain their and their loved one’s safety and health [39, 40]. This decision may potentially negatively impact the trust that has been diligently cultivated over time. Additionally, there is apprehension regarding the potential for abuse of power.

The current study was designed to explore individuals’ decisions to download VA and was based on the hypotheses that attitudes towards GT will mediate the associations between (a) trust in the healthcare system and VA downloading and (b) the perceived COVID-19 threat and VA downloading. Our findings indicate that attitudes toward GT mediate these associations, suggesting that both public trust levels and the perceived threat contribute to more favorable attitudes toward GT and a higher willingness to download VA. These findings are important for developing strategies to encourage the public to adhere to governmental policies during health crises.

### Trust and public attitudes toward GT

The overall level of trust in the healthcare system and mean attitudes toward GT among the current sample were moderate. Higher trust was associated with more favorable attitudes toward GT. These results are in line with previous studies [29, 41, 42]. Approximately half of our respondents perceived GT as an invasion of their privacy and agreed or strongly agreed with the statement that the government uses the data gathered for other purposes, and only about a quarter of the respondents reported that GT increases their sense of security. Studies suggest that in order to create supportive attitudes toward the use of digital epidemiology, a trusted public healthcare provider should be involved in developing and deploying these apps, as trust levels may be influenced by the transparency and availability of health-related information delivered to the public [43–45].

Van Velsen and his colleagues [41] showed that trust in the healthcare system might influence the public’s intentions of using digital governmental technologies. Furthermore, a recent systematic review focusing on public acceptance of digital contact-tracing apps during the COVID-19 pandemic found lower acceptance among respondents who displayed lower trust levels [45].

### Perceived threat and public attitudes towards GT

According to our findings, the greater a perceived threat is, the more positive attitudes toward GT are. Previous studies showed that perceived threat predicts attitudes [46, 47]. Therefore, it is assumed that during an emergency in which people consider their health to be seriously threatened, they may be willing to perform actions that they would not agree to perform routinely.

The association between perceived threat and attitudes toward civil rights has been studied mainly in the context of terrorism. These studies show that people are more likely to exchange their freedoms for security when under threat [39, 40]. Wnuk and her colleagues [48] demonstrated that perceived threat significantly predicts attitudes toward surveillance technologies and suggested that the realistic perceived threat to people’s health during the COVID-19 pandemic significantly predicted acceptance of potentially helpful yet controversial technologies.

### Downloading VA: adoption patterns and determinants

Most participants in this study had not downloaded VA (the Protector app), and the VA downloading rates that we found fell far short of those in other countries. In a study conducted in Singapore [49], approximately half of the participants reported downloading VA (the TraceTogether app); similar percentages were measured in England [50]. The results of a cross-country analysis demonstrate broad support for VA download [4], with approximately 75% of respondents across all countries reporting they “would probably or definitely download the contact-tracing app.” In all countries, only a minority of respondents said they would not have the app installed on their phones.

Our analysis suggests that downloading VA positively correlates with more favorable attitudes toward GT. Hence, individuals who downloaded VA were less critical of GT than non-downloaders. Amann and her colleagues [51], examining how German national newspapers reviewed the launching of the local governmental tracing app, concluded that voluntariness is an important aspect of compliance with data protection regulations.

According to our findings, downloading VA is also associated with ethnicity and religiosity. Arab participants downloaded the app more than Jewish ones. This is somewhat surprising because socially disadvantaged people, such as minority groups, tend to be less accepting of the use of tracing applications [21]. A possible explanation may be that the Israeli app Protector was available in several languages, including Arabic. Linguistic accessibility appears to have enabled and encouraged its use. Another explanation may be the stress level of the Arab population during the pandemic, possibly leading to a greater willingness to download VA [32]. Moreover, since GT had already been put into use, the VA download may have provided a sense of control.

VA download was significantly higher among secular and partly religious respondents than among religious respondents. This can be explained by the fact that many ultra-Orthodox Jews oppose innovation and most do not use smartphones [52, 53]. According to the Israel Central Bureau of Statistics [54], the ultra-Orthodox subgroup constitutes approximately 14% of the Israeli population. Therefore, the pro-VA strategy used and the efforts to trace contacts among them should be employed differently. In order to increase compliance among this population, it is crucial to recruit community leaders and design culturally adapted solutions.

Dealing with the COVID-19 pandemic began in a state of emergency requiring quick, decisive responses. Over time, the ongoing morbidity led to a change of perspective which entailed a long-term view of the situation, in which different ethical considerations must be weighed. Government surveillance must maintain the ethical principle of avoiding harm to individuals’ rights to privacy and autonomy [1]. While specific actions, such as GT, may be ethically justified during a crisis, they may become disproportional and unacceptable during routine times [54].

The current study is not without limitations. One limitation is its cross-sectional design, which rules out causal inferences among the research variables. Moreover, the data were collected using a self-reporting online questionnaire, a method that may have been susceptible to selection and recall bias. To minimize such bias, we assigned the data collection to a well-established survey company that has a large pool of respondents. This company takes comprehensive measures to mitigate selection bias by using quota sampling and restricting the number of times a person can participate in a survey during a defined period.

Another limitation concerns the non-use of smartphones, particularly among the ultra-Orthodox population [55]. People who eschew the smartphone cannot download the VA and may also differ in terms of study variables. A complementary qualitative study exploring the Israeli public’s perspectives and choices may shed light on additional aspects not examined in the current study.

## Conclusion

Public health values emphasize the importance of social solidarity in promoting the entire population’s health. At times, public health considerations may prioritize the common good over individual interests. It is, therefore, essential to consider ethical standards that balance these values with individual human rights [56].

The current study demonstrates that public attitudes toward GT mediate the association between public trust and the willingness to download VA, as well as between the perceived COVID-19 threat and the download decision. This indicates that more favorable attitudes toward GT are associated with greater cooperation in downloading VA. The results uncover two key paths to promote VA downloads: one involves enhancing the perception of threat, which can inadvertently harm public health, particularly in terms of stress and well-being [57]. The second path underscores the critical role of public trust. Establishing trust with the public is essential to encourage voluntary actions that ultimately benefit public health. Achieving and maintaining the public’s trust necessitates the addressing of concerns regarding the potential misuse of government power and the fostering of an environment conducive to voluntary participation and engagement. Finally, the findings highlight the importance of considering cultural and demographic factors in the deployment of digital health solutions.

## Electronic supplementary material

Below is the link to the electronic supplementary material.

Supplementary Material 1

## Data Availability

Data and materials are available upon request from the corresponding author.

## Abbreviations

CDC: Centers for Disease Control and Prevention
GT: Governmental tracing
ISA: Israel Security Agency
VA: Voluntary Application
WHO: World Health Organization
