# Supplementary material for: An individual’s trust in government is a major determinant in the decision to voluntarily join a public health initiative
Source: Isr J Health Policy Res. 2025 Feb 14;14:9. doi: 10.1186/s13584-025-00671-x (PMC11829482; doi:10.1186/s13584-025-00671-x)
Supplement: Supplementary file 1 — Supplementary Material 1 [file 13584_2025_671_MOESM1_ESM.docx]

**Supplementary material**

**Supplementary Table 1. Coefficients for the two mediation models (N = 741)**

| Dependent variable (R^2^) | Independent variable | B (SE) | p | 95%CI |
| --- | --- | --- | --- | --- |
| Model 1 |  |  |  |  |
| Attitudes toward GT (.13) | Trust | 0.34 (0.03) | <.001 | 0.27, 0.41 |
|  | Ethnicity | 0.21 (0.08) | .009 | 0.05, 0.37 |
|  | Religiosity | -0.05 (0.08) | .499 | -0.21, 0.10 |
|  | Marital status | -0.07 (0.07) | .334 | -0.21, 0.07 |
|  |  |  |  |  |
| Downloading VA (.17) | Trust | 0.04 (0.07) | .587 | 0.90, 1.20 |
|  | Attitudes toward GT | 0.82 (0.10) | <.001 | 0.62, 1.02 |
|  | Ethnicity | -0.49 (0.19) | .009 | -0.87, -0.12 |
|  | Religiosity | 0.74 (0.20) | <.001 | 0.34, 1.14 |
|  | Marital status | -0.21 (0.17) | .203 | -0.54, 0.12 |
|  |  |  |  |  |
| Model 2 |  |  |  |  |
| Attitudes toward GT (.08) | Perceived threat | 0.27 (0.04) | <.001 | 0.20, 0.34 |
|  | Ethnicity | 0.21 (0.08) | .012 | 0.05, 0.37 |
|  | Religiosity | -0.15 (0.08) | .074 | -0.31, 0.01 |
|  | Marital status | -0.10 (0.07) | .163 | -0.25, 0.04 |
|  |  |  |  |  |
| Downloading VA (.16) | Perceived threat | 0.02 (0.09) | .784 | -0.15, 0.20 |
|  | Attitudes toward GT | 0.73 (0.10) | <.001 | 0.54, 0.92 |
|  | Ethnicity | -0.52 (0.19) | .006 | -0.89, -0.15 |
|  | Religiosity | 0.72 (0.20) | <.001 | 0.32, 1.12 |
|  | Marital status | -0.23 (0.17) | .170 | -0.56, 0.10 |
